# Real-Time Standard Analysis of Disease Investigation (SADI)—A Toolbox Approach to Inform Disease Outbreak Response

**DOI:** 10.3389/fvets.2020.563140

**Published:** 2020-09-29

**Authors:** Paul Bingham, Masako Wada, Mary van Andel, Andrew McFadden, Robert Sanson, Mark Stevenson

**Affiliations:** ^1^Diagnostic and Surveillance Services Directorate, Operations Branch, Ministry for Primary Industries, Wallaceville, New Zealand; ^2^EpiCentre, School of Veterinary Science, Massey University, Palmerston North, New Zealand; ^3^AsureQuality, Palmerston North, New Zealand; ^4^Faculty of Veterinary and Agricultural Sciences, Melbourne Veterinary School, University of Melbourne, Parkville, VIC, Australia

**Keywords:** decision support tool, outbreak investigations, outbreak response, real-time analyses, centralised data warehouse, big data, foot-and-mouth disease

## Abstract

An incursion of an important exotic transboundary animal disease requires a prompt and intensive response. The routine analysis of up-to-date data, as near to real time as possible, is essential for the objective assessment of the patterns of disease spread or effectiveness of control measures and the formulation of alternative control strategies. In this paper, we describe the Standard Analysis of Disease Investigation (SADI), a toolbox for informing disease outbreak response, which was developed as part of New Zealand's biosecurity preparedness. SADI was generically designed on a web-based software platform, Integrated Real-time Information System (IRIS). We demonstrated the use of SADI for a hypothetical foot-and-mouth disease (FMD) outbreak scenario in New Zealand. The data standards were set within SADI, accommodating a single relational database that integrated the national livestock population data, outbreak data, and tracing data. We collected a well-researched, standardised set of 16 epidemiologically relevant analyses for informing the FMD outbreak response, including farm response timelines, interactive outbreak/network maps, stratified epidemic curves, estimated dissemination rates, estimated reproduction numbers, and areal attack rates. The analyses were programmed within SADI to automate the process to generate the reports at a regular interval (daily) using the most up-to-date data. Having SADI prepared in advance and the process streamlined for data collection, analysis and reporting would free a wider group of epidemiologists during an actual disease outbreak from solving data inconsistency among response teams, daily “number crunching,” or providing largely retrospective analyses. Instead, the focus could be directed into enhancing data collection strategies, improving data quality, understanding the limitations of the data available, interpreting the set of analyses, and communicating their meaning with response teams, decision makers and public in the context of the epidemic.

## Introduction

Concurrent with globalisation and cross-border movements, the opportunity for the emergence of new infectious pathogens in a country has increased substantially (1, 2). Some transboundary animal diseases important for food safety, international trade and livestock production, such as foot-and-mouth disease (FMD), highly pathogenic avian influenza (HPAI), and African swine fever (ASF) can spread rapidly and require a prompt and intensive response if eradication is to be achieved. However, disease eradication responses are usually resource intensive, costly and may not be justified for some diseases. Following discovery of the index case, the competent authority may decide to respond to a disease outbreak by undertaking a control or eradication program. Strategies for disease control or eradication, as well as important factors to consider before embarking on such programs, have been documented (3).

When trying to follow the course of an epidemic and judge the effects of control measures, the routine analysis of up-to-date data, as near to real time as possible, is essential to allow objective assessment of the patterns of disease spread, assessment of the effectiveness of control measures, and the formulation of alternative control strategies (4). This (often iterative) process is rarely formally documented in the published literature, but examples can be found in the United Kingdom's response to bovine spongiform encephalopathy from 1986 to 2012 (5) and the outbreak of FMD that occurred in 2001 (6, 7).

Responses to animal health epidemics increasingly deal with “big data”. Some of the challenges of dealing with big data are encompassed in the often described four Vs: volume (relatively large datasets), velocity (the speed that new data is accumulated), variety (integration of multiple sources of data), and veracity (data typically needs accuracy checking, or cleaning) (8–10). Additional to the four Vs described above, animal health responses typically have an additional V: very short time frames.

Data sources for responses typically include laboratory results from traditional and molecular diagnostic methods, animal movement records sourced from national animal movement databases or farmer records, questionnaire interview data, targeted risk based sampling, and opportunistic sampling data. All these data must be underpinned by national farm or animal level demographic datasets.

The key to achieving real-time assessment of ongoing control measures is the presence of a decision support tool, i.e., a data warehouse capable of integrating all data sources and with functions of automated analyses and reporting tailored to the outbreak, and available as early as possible. The components of decision support tools that can be used in animal health have been previously described (2, 11, 12). These should be designed and set up, wherever possible, during non-response (peace time) periods to address the challenges described, particularly ensuring internal validation of the tool, and understanding the limitations and biases in required datasets. Such tools should ideally be centralised, contain relational databases all-inclusively, and ensure that any updates in the system be reflected instantly. Animal disease response activities necessitate that the tools used for management or analysis of data be developed within the regulatory authority. This is due to real issues around data consistency between response teams, sharing, and confidentiality (13). The software platform plus analytics needs to have both utility and usability in that the analyses can be run frequently and in real time, the interface allows new users to quickly learn and use the tool, and this in turn frees up limited numbers of epidemiology personnel to interpret the analyses and improve data quality. The data and analytics should be accessible to epidemiologists, for exploration and augmentation as required.

As part of New Zealand's biosecurity preparedness, a tool named real-time Standard Analysis of Disease Investigation (SADI) has been developed for performing standardised analyses during disease outbreaks. Our focus was development of a data warehouse together with a standardised set of analyses for use by epidemiologists seconded into a large FMD response, should one occur. Their usual role outside of the response may not include infectious disease epidemiology or the use of programming languages. Therefore, SADI has a simple, user-friendly interface so that the focus can be on improving data quality, understanding the limitations of the data available and interpreting the set of analyses and their meaning in the context of the epidemic. Our goal was to standardise and automate the analyses and increase the time available to interpret and communicate outbreak metrics and patterns. SADI has been used for domestic and international training of epidemiologists in biosecurity outbreaks, and in the ongoing *Mycoplasma bovis* eradication programme since 2017. The aim of this paper is to describe SADI using a hypothetical FMD outbreak as an example. FMD was chosen because it is the major threat to New Zealand livestock industries due to its high contagiousness and significant economic impact. SADI could be modified and applied to other diseases, for example, HPAI or ASF.

## Materials and Methods

### General Description

SADI was developed as a customised project within an application, Integrated Real-time Information System (IRIS)^1^ (EpiSoft Ltd, New Zealand). The following sections describe the structure of SADI in terms of the platform, data, and analysis.

### Platform

Integration, data management, and analysis were conducted within IRIS.

IRIS is a secure, web based, data management application, based on a dynamic data storage system. All data administration and processing are achieved via a web portal. Multiple portals can be added and customised according to organisational needs. Data can be accessed from any remote location with an internet connection, using any device with a browser. Data storage uses the adaptive object model (14), and access is restricted to authorised personnel using role-based access control.

The application can import and store virtually any type of data including, but not limited to text, images, vector, and raster spatial data. Data are imported into the system using industry standard formats. The framework allows third party applications to communicate with it via web services. Data can then be filtered, sorted and grouped to create customised views. Project managers have the flexibility to change and modify data schemas as their requirements change over time.

**Figure 1:**
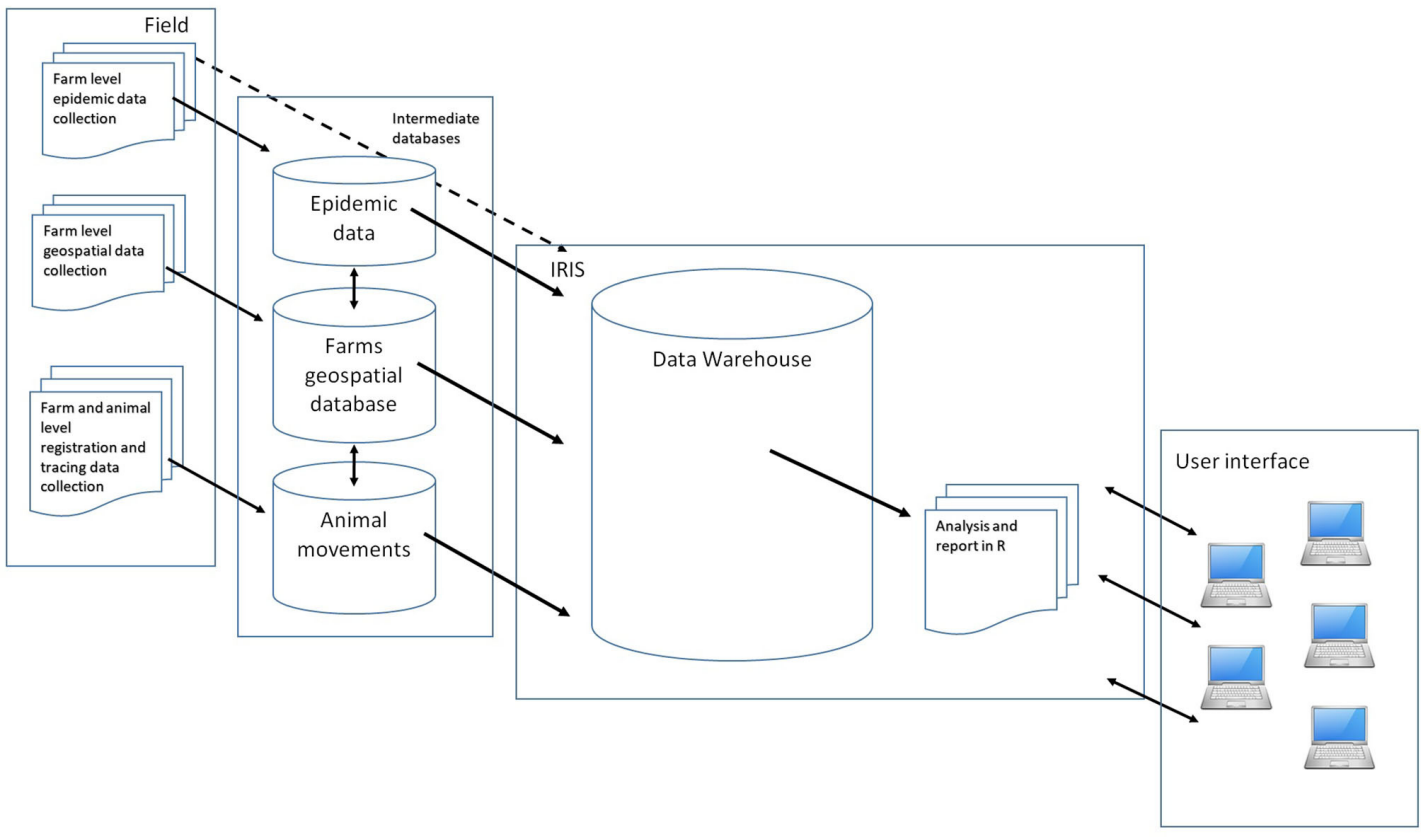
Schematic of the Standard Analysis of Disease Investigation (SADI), showing how relevant field data are collected, integrated, analysed, and reported to the response intelligence using Integrated Real-time Information System (IRIS).

Figure 1 shows how existing databases and response specific field data came together for analyses within SADI using IRIS as the data warehouse and analytical toolbox.

**Figure 2:**
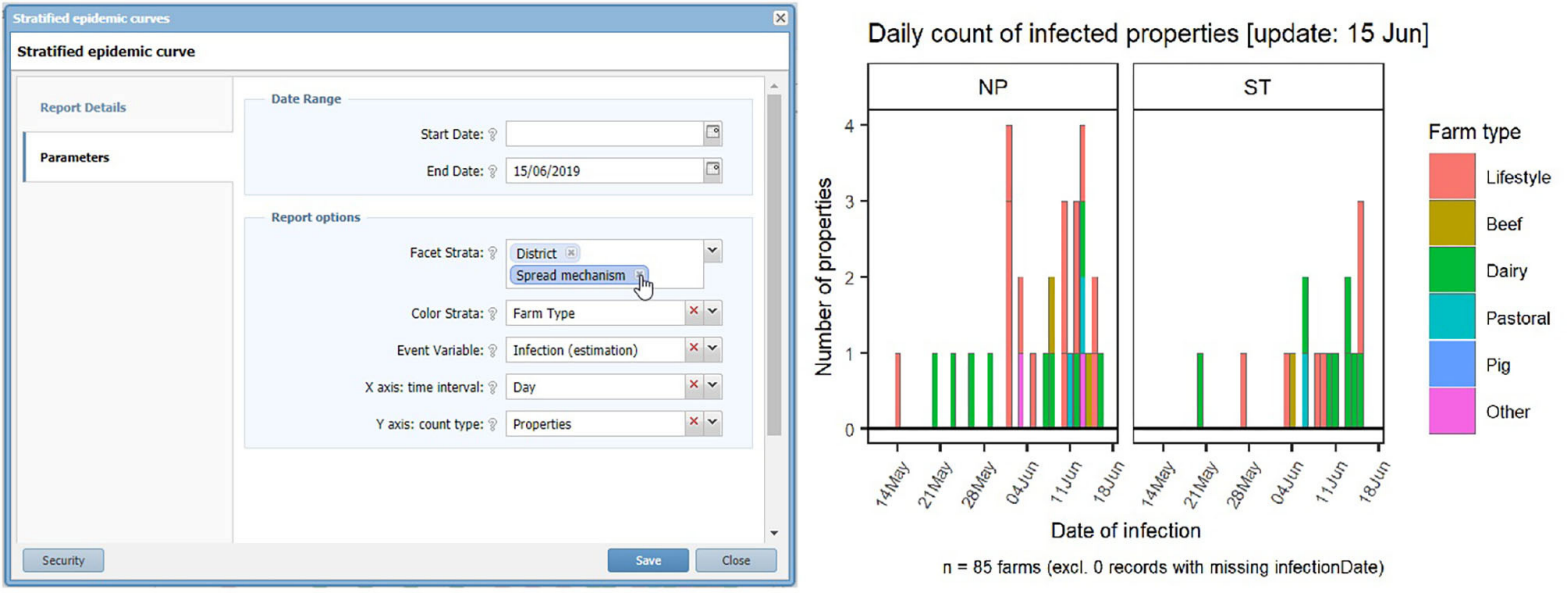
The wizard style user interface of the Standard Analysis of Disease Investigation (SADI) within Integrated Real-time Information System (IRIS). This example is the window allowing selection of parameters for a stratified epidemic curve. Using selection and drop-down boxes the analyst can manipulate the input parameters.

The reporting engine is powered by the R statistical software^2^ (R Core Team, Vienna, Austria). However, IRIS has a wizard style user interface making the running of any R report a relatively simple exercise for non-proficient R users. A typical example of the wizard style user interface, which sits between the user and the R code, is shown in Figure 2. Key parameters can be changed easily and the analysis re-run to quickly explore patterns in the data.

Reports can be scheduled to be automatically updated as frequently as required, for example every 24 h, to ensure that the interpretation and the assessments are made based on the most up-to-date data.

### Datasets

For our FMD epidemic scenario, three datasets are required to perform the standardised analyses: an outbreak dataset, a tracing dataset, and a population dataset. They are linked by a common field, a unique farm identifier. For other types of diseases, additional datasets (e.g., laboratory data or slaughterhouse data) could be included, if required. The data frame can flexibly be modified, such as accommodating additional fields or using animals as epidemiological units. The database was designed so data fields were comprehensive without redundancy, to avoid data inconsistency within the system.

The outbreak dataset would be supplied from field investigations performed on confirmed infected farms (affected farms with infected animals present). This data for individual farms can be entered into the platform directly in the field, using for example, a handheld device. The data for multiple farms can also be imported into the platform after transcribing field questionnaire data into a comma separated value (CSV) file. Alternatively, the data can be imported indirectly from an external response database. The design of the outbreak data is described in Table 1.

**Table 1:** The design of the outbreak data for the Standard Analysis of Disease Investigation (SADI).

**Field name**	**Field description**	**Data format**
FarmID	Unique farm identifier^†^	String
InfectedNo	Infected place number	Integer
NoticeNo	Legal notice number	Integer
PublicReport	If the infection is publicly reported	Boolean
SurveillanceType	Surveillance type which identified the farm	Categorical (public report/surveillance zone/tracing)
VisitDate	First visit date	Date
InfectionDate	Estimated infection date	Date
LesionAge	Age of oldest lesion seen	Numeric
ClinicalDate	Date of onset of clinical signs^•^	Date
DiagnosisDate	Date of diagnosis or laboratory confirmation	Date
SlaughterDate	Date when slaughter is completed	Date
DisposalDate	Date when disposal is completed	Date
CleanDate	Date when cleaning and disinfection is completed	Date
SpreadMechanism	Likely spread mechanism	Categorical (movement/contiguous property/associated property/local spread^§^/ other)
SourceFarm	Likely source farm (if known)	String
DairyInfected	Number of clinically infected dairy cattle	Integer
BeefInfected	Number of clinically infected beef cattle	Integer
SheepInfected	Number of clinically infected sheep	Integer
PigInfected	Number of clinically infected pigs	Integer
GoatInfected	Number of clinically infected goats	Integer
DeerInfected	Number of clinically infected deer	Integer
UpdateDate	Date when the data were last updated	Date

† *Common farm identifiers to link with the other datasets, i.e., tracing data and population data*.

‡ *This field can automatically be filled in based on the visit date and oldest lesion age*.

§ *Local spread includes direct and indirect spread mechanisms such as unknown movements, general local farmer activities, aerosol or wind spread, fence contacts, and potentially wildlife spread*.

As no actual FMD outbreak data was available in New Zealand during the development of this tool, the authors simulated hypothetical outbreak data from the New Zealand Standard Model for FMD (NZSM) (15–17).

The tracing dataset required is described in Table 2. During an outbreak, this dataset would be sourced as part of the epidemiological interview and from national livestock traceability systems. As traceability systems are usually focused on live animal movements, both of these methods would be used and possibly others to collect a comprehensive list of possible disease conveyors. In New Zealand, the three main tracing data sources would be epidemiological interview; the National Animal Identification and Tracing (NAIT) system^3^ (OSPRI, Wellington, New Zealand) (which at the time of publication covers cattle and deer); and the Animal Status Declaration (ASD) system which is a hard copy traceability system covering all FMD susceptible species^4^ (Ministry for Primary Industries; MPI, Wellington, New Zealand).

**Table 2:** The design of the tracing data for the Standard Analysis of Disease Investigation (SADI).

**Field name**	**Field description**	**Data format**
TraceID	Trace event unique identifier	String
TraceFarmID	Unique identifier^†^ of a farm being traced	String
LinkedPlaceID	Unique identifier^†^ of a farm identified as being connected with the traced farm	String
MovementDate	Movement date	Date
MovementDirection	Movement direction	Categorical (forward/backward)
Conveyor	Conveyor type	Categorical (live animal/carcass/genetic material/dairy tanker/other animal product/non-animal product/fomites/other)
Risk	Risk level based on the assessment of the conveyor type	Categorical (low/medium/high/unknown)
CountAnimals	Count of animals moved (if conveyor type is animals)	Integer
DataSource	Data source	Categorical (interview/NAIT^‡^/ASD^§^)
UpdateDate	Date when the data were last updated	Date

† *Common farm identifiers to link with the other datasets, i.e., outbreak data and population data*.

‡ *National Animal Identification and Tracing system*.

§ *Animal Status Declaration system*.

Again, as no actual infected farms were available during development of SADI, tracing data were simulated by the NZSM.

The population dataset needs to be collected prior to outbreak responses and updated regularly, as part of disease preparedness. For New Zealand livestock population, the data were sourced from AgriBase® which is a commercially available, comprehensive, spatially explicit, farm level, demographic database, describing commercial and non-commercial properties holding production animals in New Zealand (18, 19). The design of the population data is shown in Table 3. Details of farms such as the names of farm owner/manager and contact details are not required to perform standardised analyses but are required for other operational response purposes. The access to these data fields can be restricted to authorised persons only.

**Table 3:** The design of the population data (sourced from AgriBase™) for the Standard Analysis of Disease Investigation (SADI).

**Field name**	**Field description**	**Data format**
FarmID	Unique farm identifier	String
Owner^†^	Farm owner	String
Manager^†^	Farm manager	String
Phone^†^	Contact phone number	String
Email^†^	Emil address	String
Address^†^	Farm address	String
District	Farm district	Categorical (67 territorial local authorities)
PostCode	Farm postal code	Integer
X coordinates	The longitude of the farm centroid	Numeric
Y coordinates	The latitude of the farm centroid	Numeric
FarmType	Types of farms by animal species, production or management, which are of epidemiological importance	Categorical (dairy/beef and sheep/lifestyle/pigs)
Dairy	Number of dairy cattle	Integer
Beef	Number of beef cattle	Integer
Sheep	Number of sheep	Integer
Goat	Number of goats	Integer
Pig	Number of pigs	Integer
Deer	Number of deer	Integer
UpdateDate	Date when the data were last updated	Date

† *Data fields that are required for operational response purposes and with restrictive access*.

### Analytics

A set of standard analyses was collected by reviewing literature or gathering opinions from MPI staff. The use of these analyses is for summarising and visualising data for response or tracing teams; describing the current situation for informing intelligence and public awareness; building hypotheses about risk factors; or measuring efficiency and effectiveness of the ongoing response efforts.

For each analysis, a report template composed of a variable table, data queries and an R code was created within SADI. A variable table listed a set of input parameters that were necessary for conducting this particular analysis and would accommodate parameter values entered by an analyst, as shown in Figure 2. Data queries specified the data fields necessary for conducting this particular analysis. Based on these queries, the most up-to-date datasets were drawn from the internal database in SADI each time the analysis was carried out. An R script was developed, which would process the datasets using the input parameter values, analyse, and output a report in image (png, jpg, svg, etc.), web page (hypertext markup language; HTML), or map (keyhole markup language; KML) format. Data manipulation and visualisation was commonly conducted using R packages reshape2 (20), plyr (21), and ggplot2 (22).

This set of analyses were programmed to run at an optimal interval (e.g., 24 h) so that the updated analyses would reflect new data values that were entered after the last analyses.

## Results

The list of analytic reports that were collected for the use of FMD outbreak response and incorporated in SADI is shown in Table 4. There were 16 reports, of which 12 could be used for assessment of response effectiveness and efficiency, seven for informing intelligence and public awareness, five for hypothesis building and four for assisting tracing (some reports were counted multiple times).

**Table 4:** Summary of analytic reports incorporated in the Standard Analysis of Disease Investigation (SADI) for a foot-and-mouth disease (FMD) outbreak response, with function, input parameters, dependent data* (O, outbreak data; T, tracing data; P, population data), and primary use.

**Name**	**Function**	**Input parameters**	Data^*^	**Primary use**
Individual farm timeline	Timeline of a particular infected farm with backward and forward traced movements to/from this farm with the dates and movement details, mapped on a line which represents the course of infection of the farm.	Farm identifier, incubation period range, maximum possible sub-clinically infectious period	O, T	Tracing
Response timeline	Temporal trend of the duration taken to complete each response activity (detection/depopulation/disposal/cleaning) for each infected farm on the scale of calendar date.	Date range, farm sorting criteria	O	Efficiency assessment
Response efficiency boxplot	Temporal trend of the duration taken to complete each response activity.	Strata, response activity (detection/depopulation/disposal/cleaning), time interval	O	Efficiency assessment
Network map	A map showing the point location of a particular infected farm and the farms associated with this infected farm identified from backward or forward tracing. The map was generated as a Keyhole Markup Language (KML) format, allowing it to be zoomed in and out or display the details of farms or movements by clicking the features.	Farm identifier, incubation period range, maximum possible sub-clinically infectious period	O, T, P	Tracing, intelligence/awareness
Outbreak map	A map showing the point locations of all identified infected farms and at-risk farms within a buffer (e.g., 3 km protection zone) or traced farms. The map was generated as a Keyhole Markup Language (KML) format, allowing it to be zoomed in and out or display the details of farms or movements by clicking the features.	Buffer width, maximum possible sub-clinically infectious period	O, P	Tracing, intelligence/awareness, efficiency assessment
Stratified cumulative epidemic curve	Temporal change in the cumulative counts of infected farms (or animals) by estimated infection date or date of diagnosis, with an option to stratify by potential risk factors (e.g., district, farm type, spread mechanism).	Strata, infection stage (infected/diagnosed), count unit (animals/farms)	O	Intelligence/awareness, hypothesis building, efficiency assessment
Stratified epidemic curve	Counts of newly infected farms (or animals) per unit time by estimated infection date or date of diagnosis, with an option to stratify by potential risk factors (e.g., district, farm type, spread mechanism).	Strata, infection stage (infected/diagnosed), count unit (animals/farms), time interval	O	Intelligence/awareness, hypothesis building, efficiency assessment
Number of infectious farms	The total counts of infectious farms each day over time by farm states (subclinical infectious, clinical and undiagnosed, diagnosed and waiting for slaughter).	Maximum possible sub-clinically infectious period	O	Intelligence/awareness, efficiency assessment
Estimated dissemination rate (EDR)	Temporal change in the EDR for n days, calculated as the number of new cases in one time period (day i to day [i-n+1], inclusive) divided by the number of new cases in the previous time period (day [i-n] to day [i-2n+1], inclusive), based on the estimated infection date or diagnosis date. Confidence intervals around each daily EDR were derived from simulated EDRs of 99 iterations using random numbers drawn from Poisson distributions with the calculated numerator and denominator values as the mean. The analysis used an R package epiR (23).	Strata, infection stage (infected/diagnosed), base number of days (n), inclusion of loess smooth line, span for loess smooth	O	Efficiency assessment
Estimated reproduction number (R_0_)	Temporal change in R_0_ with a week window, with the given serial interval distribution. The posterior mean and 95% credible intervals for R_0_ is obtained within a Bayesian framework. The serial interval distribution was based on the parametric method with the given mean and standard deviation of the serial interval. This analysis used an R package EpiEstim (24).	The average number of days from the date of infection to when they become infectious and standard deviation	O	Efficiency assessment
Kernel smoothed density map	A map of kernel smoothed density of infected farms (expected number of farms per square km). The smoothing bandwidth was determined by the standard deviation of an isotropic Gaussian kernel. The bandwidth could be adjusted by a specified factor. This analysis used an R package spatstat (25).	Adjustment factor for the smoothing parameter	O, P	Intelligence/awareness
Nearest neighbour distance	Temporal trend of the distance from a newly infected farm to the nearest infectious farm (potential infection source), with an option to stratify by risk factors (e.g., farm type, district). Distance was computed using a package sp (26).	Strata, plot type (boxplot/histogram)	O	Hypothesis building, efficiency assessment
Areal attack rates	Temporal change in the estimated areal attack rates for each day over time, calculated as proportions of farms that became infected among all the susceptible farms located within a specified buffer from infected farms during their infectious period (27). Distance was computed using a package sp (26). Confidence intervals around the estimated areal attack rates were calculated by the exact method for probabilities using epiR package (23, 28).	Buffer width, maximum possible sub-clinically infectious period, inclusion of loess smooth line, span for loess smooth	O, P	Hypothesis building, efficiency assessment
Area under control	Temporal change in the total area size of a buffer (e.g., 3 km protection zone) around identified infected farms and the total number of susceptible premises within the buffer. The area size was computed using rgeos package (29).	Buffer width	O, P	Intelligence/awareness, hypothesis building, efficiency assessment
First day incidence	Temporal trend of the proportions of animals showing clinical signs in an infected farm on the day of first visit divided by farm type or animal species, which indicates the infectiousness of the farm in terms of forward risk potential during the period from infection to diagnosis (30). Confidence intervals for the incidence risk estimates were calculated using Wilson's approximation (31). This analysis was conducted using incursion package (32).	Strata	O	Hypothesis building, efficiency assessment
Incubation period	The distributions of the number of days since the estimated infection date until the onset of clinical signs based on the data from all identified infected farms.	Strata	O	Tracing, hypothesis building

**Figure 3:**
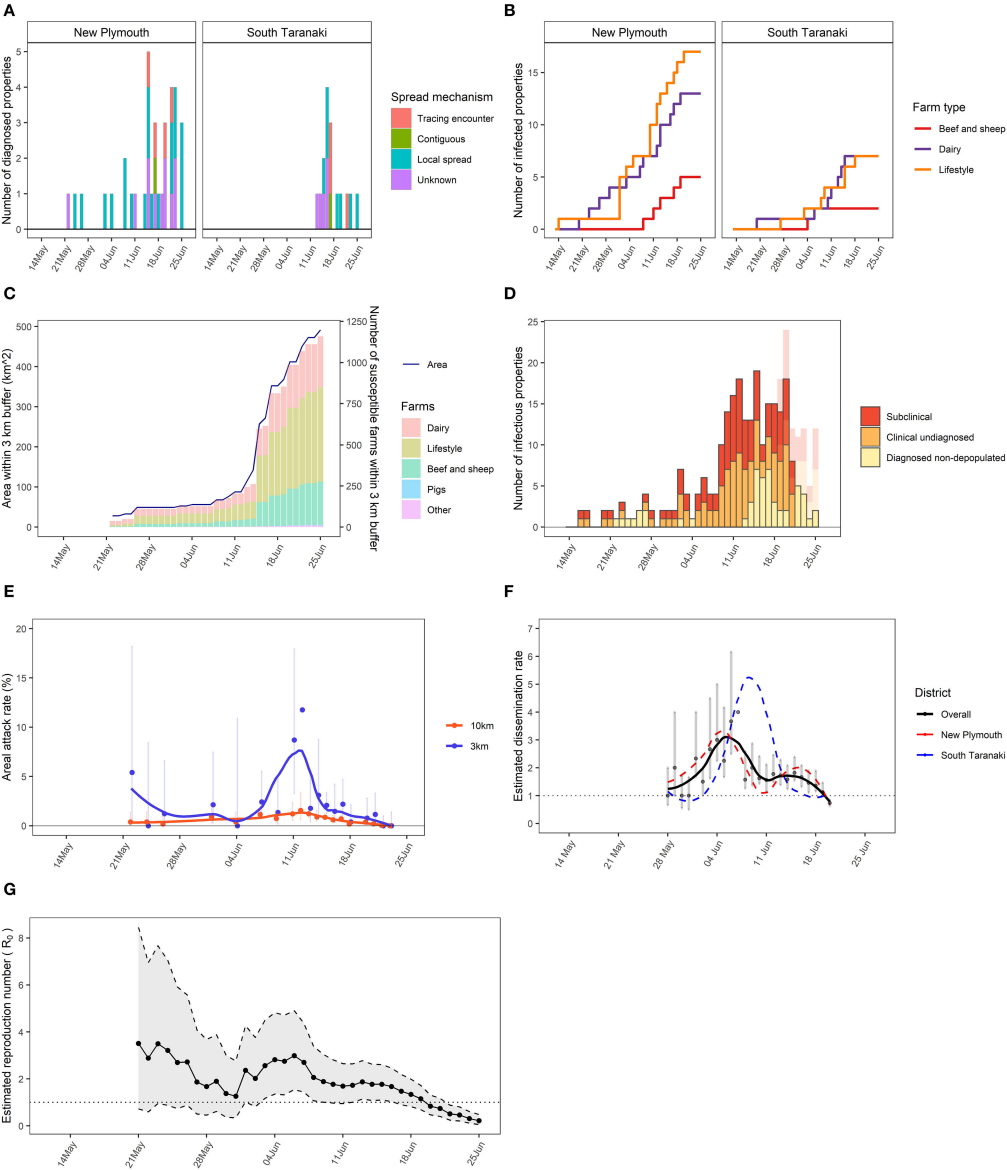
A subset reports generated by the Standard Analysis of Disease Investigation (SADI) for a hypothetical foot-and-mouth disease (FMD) epidemic, using the data available up to the date of analysis. **(A)** Stratified epidemic curve showing counts of infected farms based on diagnosis dates by district and spread mechanism. **(B)** Stratified cumulative epidemic curve showing cumulative counts of infected farms based on infected dates by district and farm type. **(C)** Area under control within a 3 km protection zone around identified infected farms over time, and numbers of susceptible farms within the area by farm type. **(D)** Total number of infectious farms each day over time that are subclinical, clinical and undiagnosed or diagnosed and non-depopulated, with the bars in transparent colours showing the actual numbers using the complete data that were obtained retrospectively. **(E)** Areal attack rates within 3 and 10 km zones, calculated as the proportions of farms that became infected among all the susceptible farms located within 3 or 10 km buffer from infected farms during their infectious period, with 95% confidence intervals, and loess smoothed trendlines. **(F)** Estimated dissemination rate (EDR) with a basis of 7 days with 95% confidence intervals and loess smoothed trendlines overall or stratified by district. **(G)** Estimated reproduction number (R_0_) over time with a week window, and 95% credible intervals.

With a hypothetical FMD outbreak scenario, infection and detection in 51 farms in New Plymouth and South Taranaki were simulated over 5 weeks. An animated figure showing the spread of this hypothetical FMD epidemic between farms is available as a Supplementary Material. A subset of reports produced 4 weeks (25 June 2019) after detection of the index case (22 May 2019) is shown in Figure 3. The stratified epidemic curves provided an indication of the temporal pattern of incidence, importance of local spread as the common spread mechanism and predominance of infection in lifestyle blocks (hobby farms) and dairy farms (Figures 3A,B). Note using estimated infection dates instead of diagnosis dates removed some of the influence of surveillance intensity after the recognition of disease (Figure 3B). The area under control showed the presence of over 1,100 susceptible farms locating within the 3 km radius protection zones, dominated by lifestyle farms (Figure 3C). The majority of infectious farms were undiagnosed farms, warranting enhanced surveillance for early detection of these farms, as well as increased capacity for depopulation (Figure 3D).

The areal attack rates showed a higher rate of secondary infection within 3 km of infected farms on the 4th weeks of the outbreak, indicating that disease mainly propagated locally (Figure 3E). Both districts had an Estimated Dissemination Rate (EDR) decreasing over time and approaching 1 at the time of the analysis (Figure 3F). If this trend continued, it would indicate that control measures were bringing dissemination of infection under control. The effective reproduction number (R_eff_) was approaching 1, which had a similar indication as the EDR (Figure 3G).

**Figure 4:**
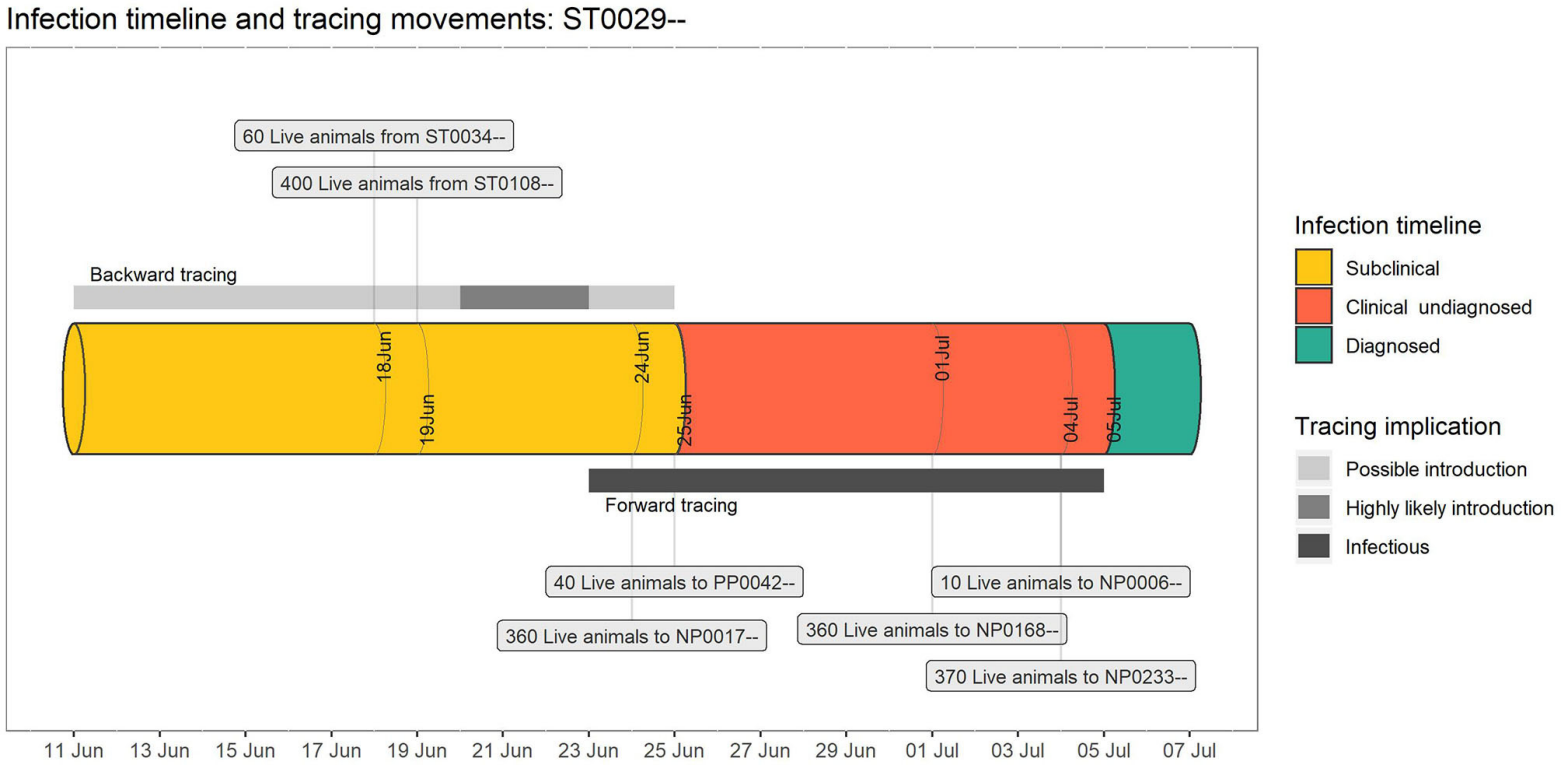
An example individual farm timeline for a hypothetical foot-and-mouth disease (FMD) outbreak, generated by the Standard Analysis of Disease Investigation (SADI). The pipe represents the timeline of a particular infected farm, with forward and backward risk windows for tracings in scaled grey bars (assuming incubation period: 2–14 days; maximum subclinical infectious period: 2 days), and the source and destination properties which supplied or received tracings associated with this property in a text box.

Figure 4 shows the timeline of a particular farm (ST0029–) that was recently diagnosed (5 July 2019). This timeline demonstrated identifying seven farms as having contacts with this farm in the potential introduction period and potential infectious period. From backward tracing, two farms were identified as the potential source of infection, whereas five farms were identified as potentially infected from this farm by forward tracing.

**Figure 5:**
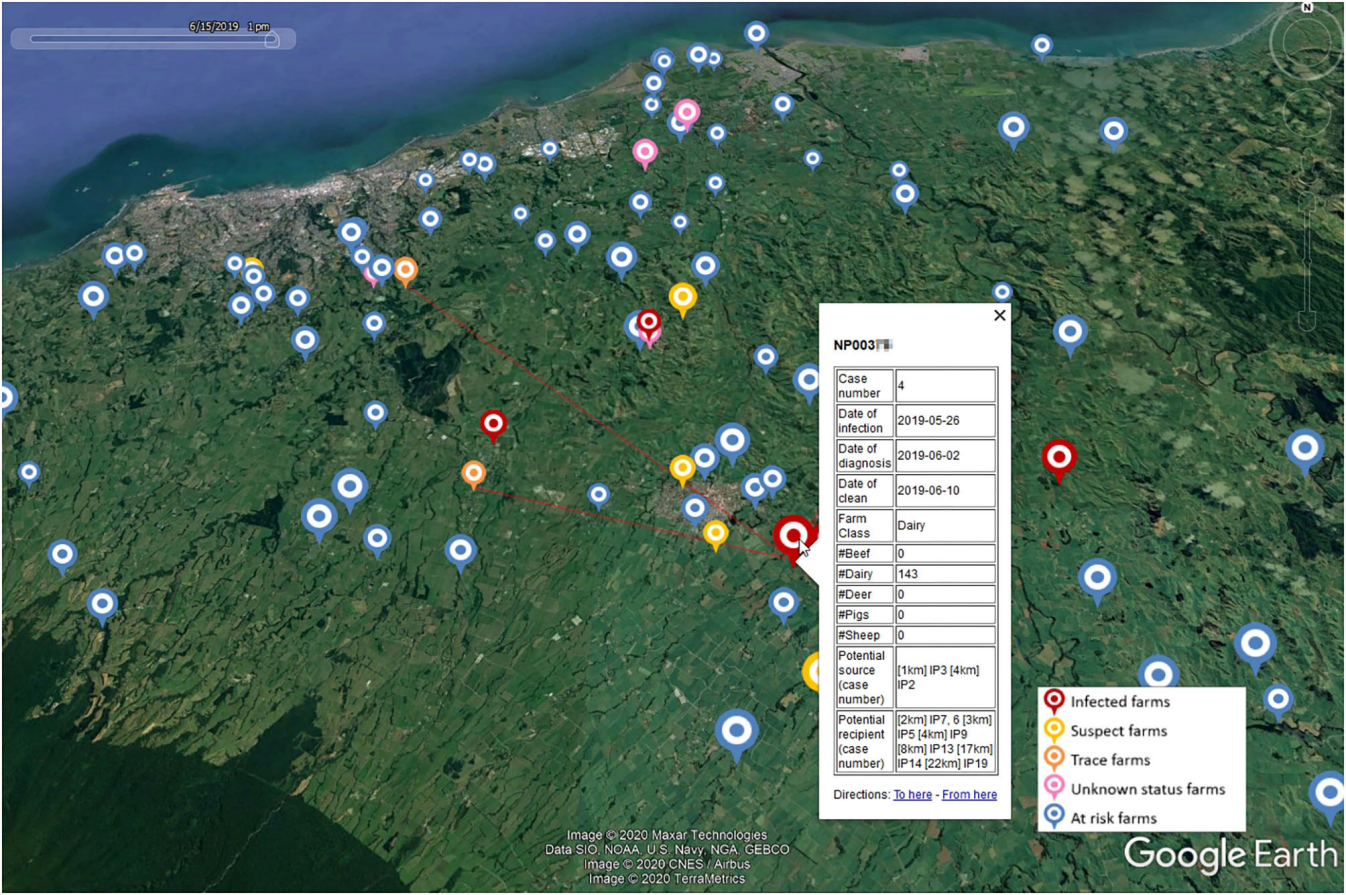
A snapshot of a network map for a hypothetical foot-and-mouth disease (FMD) outbreak on a particular date (15 June 2019) generated by the Standard Analysis of Disease Investigation (SADI). The icons represent farms, with icon colours representing farm status and icon size representing the number of animals present. Selection of a particular farm of interest (NP003xx) displays the details of this farm including all temporally plausible infection source and recipient farms by distance, and traced movements from or to this farm.

Figure 5 is a snapshot of a network map on a particular date (15 June 2019), showing the point locations of the farms in various state (infected, suspect, traced, unknown, at risk). The map also showed the details of a selected farm (NP003xx) as well as two traced movements from or to this farm. This would allow field investigators to prioritise surveillance of linked properties. Additionally, in efforts to identify risk factors associated with disease spread, network analyses could be used to select controls for case-control studies matched on time.

**Figure 6:**
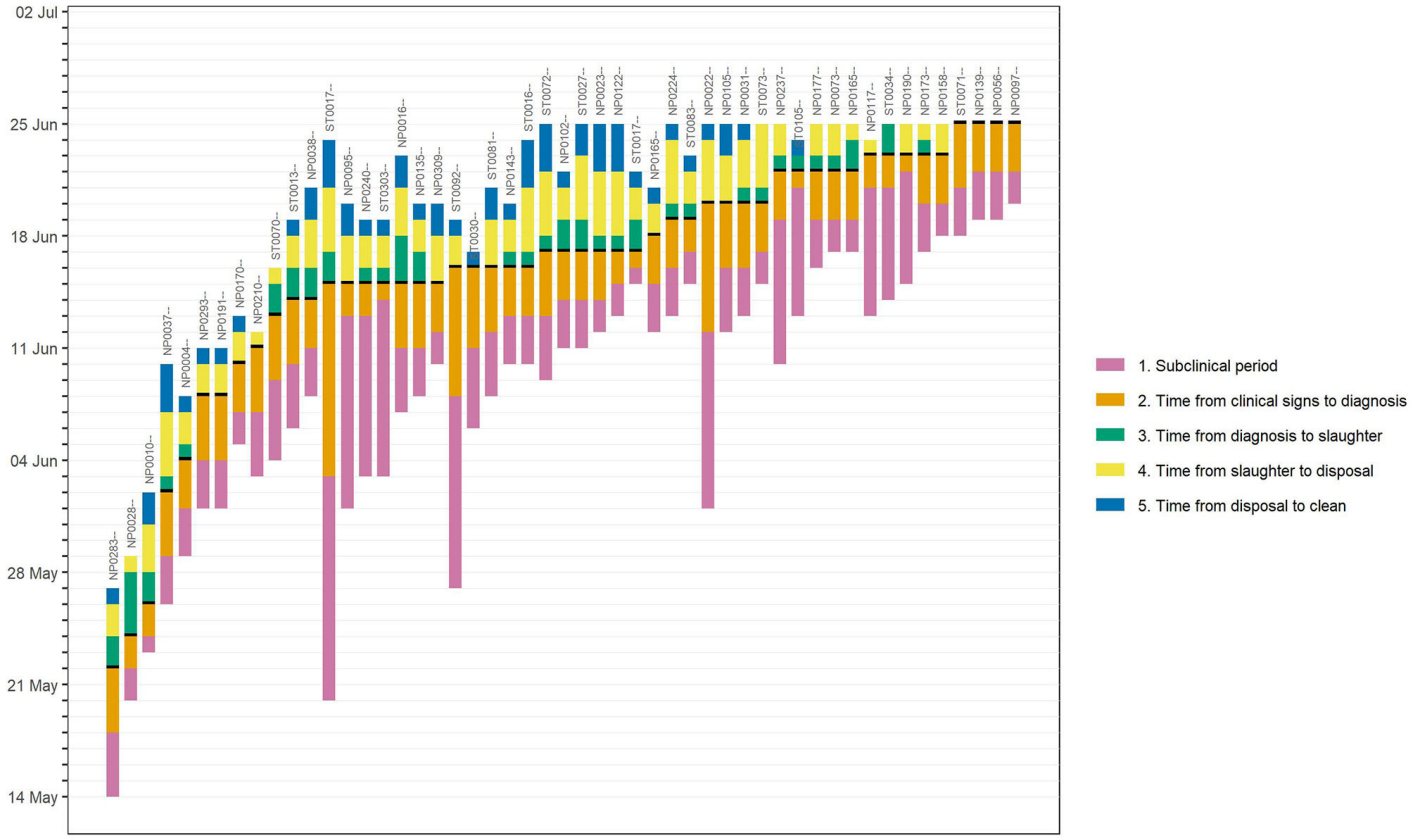
An example response timelines for a hypothetical foot-and-mouth disease (FMD) epidemic, generated by the Standard Analysis of Disease Investigation (SADI). Each bar represents the timeline of an individual infected farm. The bar length shows the infection stage or durations taken to complete each control activities (subclinical infection period; time from the onset of clinical signs to diagnosis; time from diagnosis to completion of slaughter; time from completion of slaughter to completion of disposal; time from completion of disposal to completion of cleaning and disinfection).

Response timelines depicted the timeliness of response activities for all infected properties and indicated the operational capacity of the response organisation (Figure 6). For example, long delays (8–12 days) from the onset of clinical signs to diagnoses were highlighted for three farms (e.g., ST0017–, ST0092–, NP0022–), indicating extra resources may be required to improve communication between farmers and veterinarians and increase public awareness.

## Discussion

Here we described SADI, which functions as a centralised data warehouse and performs real-time analyses during a response to an animal health epidemic. This paper demonstrates how the standardised analyses prepared in advance and largely automated, allow description of disease spread as near to real time as possible, assessment of effectiveness of response control measures and input into the formulation of new strategies. By automating the analysis steps and using a user-friendly interface, a wider group of epidemiologists can focus their time away from daily “number crunching,” or providing largely retrospective analyses. Instead, the focus can be directed toward optimisation of data collection, exploration of data quality, and quantity prior to any analysis occurring, which then (importantly) enable them to understand the limitations of the data, interpret the analyses produced and provide more immediate advice to other response teams and decision makers. Highly specialised epidemiologists and in particular those with experience with data science, R coding and disease outbreak investigation can be used to refine the analyses in place. To the best of our knowledge, this is the first time such a tool was developed for the livestock population in New Zealand.

Although the system has not been used for real FMD data, it was tested with various simulated FMD incursion scenarios through a series of internal and external workshops involving epidemiologists and programmers. These workshops have helped improving the system, detecting any misfunctions to be fixed and discussing limitations of the system. SADI has also been used for the real outbreaks of *M. bovis* in New Zealand (2017). For the *M. bovis* outbreak, additional analytical reports were developed to meet the specific needs of *M. bovis* epidemiology and response activities. The outcomes of the tool have been communicated widely among the epidemiologists, response teams, tracing teams and decision makers, and demonstrated its value in providing timely information. Particularly, SADI has shown its advantage in timeliness as well as consistency in automatically providing up-to-date reports over 2 years with minimum resource use, in comparison with other systems or the traditional manual approaches.

The ready availability of near-real time graphs, maps and models present some challenges. During a large disease outbreak, staff who are unfamiliar or undertrained, or imported foreign veterinarians may not understand the implicit biases and caveats, misrepresenting the progress of the disease control operation. It is therefore important that these reports are intuitive and clear. There is also a need for cartography standards for outbreak situation reports.

Also, the outbreak data would typically become available with a lag equivalent to the incubation period plus detection delay. Due to this lag, there is a varying extent of difference between real-time analyses using the incomplete data available on the date of analysis and the retrospective analyses using the complete data. Typically, this results in the underestimation of disease risks shortly before the date of analysis (e.g., Figure 3D). The analytic reports should be interpreted with caution or the data might be right censored prior to the date of reporting. The tool is therefore best in the hands of epidemiologists who should be involved in communicating at all levels of the programme.

For FMD, a standard set of useful analyses has been described (4). Even though most of these analyses, as well as additions, have been developed in SADI, the method described is equally applicable to most if not all epidemics and probably to all biosecurity domains (domestic animal health; plant health; marine health). As inferred above, a well thought through set of analytics specific to the disease being considered is better prepared in peace-time. Refinements can then be undertaken during an outbreak.

Large biosecurity events can occur unpredictably and can put significant, competing demands on the resources of the regulatory authority well beyond usual levels. For high impact diseases such as FMD, many countries have contingency plans in place to allow a pre-programmed set of rapid actions, and set in place a structure for decision making early in the response. This is important because the economic impacts resulting from FMD outbreaks can be enormous (33–36).

However, even with the presence of response plans, mounting an effective response to a large animal health outbreak can be challenging. Animal health professionals and in particular epidemiologists are well-suited for many roles in disease response and are usually in short supply. To compound this, new and existing staff may have no experience of the disease being controlled, may be unfamiliar with required data sources, data collection and collation methods, or the specific analyses required. Defining the data requirement, setting up data collection strategies and defining and then performing analyses all during the response is not ideal, and is an approach likely to fail.

SADI can form an integral part of the suite of intelligence tools used by epidemiologists during a response. As noted earlier, many of the data sources used in a response are common to syndromic scanning surveillance. An example would be a national farm demographic dataset which can additionally be used by an epidemic outbreak model. Multiple uses avoid development of tools for siloed applications (13).

As the volume and complexity of infectious disease data increases, professionals must synthesise highly disparate data to facilitate communication with the public and inform decision makers (13). The need for integration of data from a range of sources, into a single data warehouse for analysis is a strong argument in favour of setting up such platforms as a part of readiness between outbreaks. In this paper we have described integration of national farm demographic data, field outbreak data, and individual animal tracing datasets. There are many other possible sources of useful data including laboratory data, industry data such as milk recording at the farm level or meat processing data and vehicle tracing data. If the unit of interest for an outbreak changed from the usual farm level to the individual animal level, other existing data sources will become more common as precision agriculture progresses.

The exploration of data integration including alternative data sources is potentially valuable in augmenting the operation of the tools and improving the response efficiency. The way that data are generated has changed radically over the last 30 years, mainly as a result of the emergence of electronic methods of measuring, recording, storing, and distributing data (1). Syndromic surveillance systems are becoming increasingly important tools to monitor disease outbreaks by making use of available data (37). Integrating such systems with SADI may help early detection of disease and a prompt start of response activities. While many of these data sources may be protected by legislation from use during “peacetime” surveillance, they could become available during biosecurity responses. The custodians of these datasets may be willing to help ensure integration of data as contingency planning to protect their industries.

While the amount of data potentially available for integration and analysis continues to increase, the development of suitable analytical tools for converting this raw data into useful knowledge has been much slower (9, 38).

Key themes in the development of effective visualisation and analytical tools for infectious disease epidemiology have been described (13). These include: the importance of knowledge regarding user needs and preferences, the importance of user training and the integration of the tool into routine work practices, understanding the complications associated with use of visualisation, the role of user trust and organisational support in the ultimate usability and uptake of these tools. The paper also noted that individual tools and datasets are rarely sufficient, even for local decision making. Therefore, it is important that the systems under development are tested well in advance by a group of potential users during training exercises, and improvements made through feedback from them. Also, interoperability of tools, data sharing and integration, and sustainability of the tools are important goals that should factor into the design of tools.

Additional to these themes, analytics which are targeted to the objectives of a response and best approaches used in an animal control or eradication program are essential. For example to control an epidemic of FMD, it is essential to understand the mechanisms by which FMD virus is being spread (39). A substantial amount of research has been conducted and described on methods for analysing animal health epidemic data (1, 4, 11, 40–45). However, many of the analyses described were conducted retrospectively and therefore were not available to decision makers in real time during the outbreak. Disease spread patterns are complex, affected by the underlying susceptible population, climate and geography, and the priorities of the stakeholders change over time and vary by country. Therefore, there is no established best strategy that works for every epidemic. The key to successful decision making is based on a good understanding of the disease in question, based on the timely analysis of the field data.

SADI with simulated outbreak datasets can be used as training materials. The authors have produced simulated FMD outbreaks using InterSpread Plus (46) with deliberately introduced sub-optimal response parameters. The subsequent simulated datasets have then been analysed during training exercises exploring response effectiveness and efficiency. By not dedicating large amounts of time to performing the analyses but rather to interpreting them, rapid understanding of the epidemic and response effort is achieved as well as an appreciation that different analytics are useful at different phases of the epidemic. Conversely, during a true disease outbreak, the standard set of analyses includes response specific parameters for the simulation model. The model would then be tuned to the particular strain of FMD. A set of economic analytics and resource calculators would be a next logical step.

## Data Availability Statement

The datasets presented in this article may be available on request. Restrictions apply to the availability of these data, which were used under license for this study.

## Author Contributions

All authors have contributed to the work and approved of publication.

## Conflict of Interest

RS was employed by the company AsureQuality. The remaining authors declare that the research was conducted in the absence of any commercial or financial relationships that could be construed as a potential conflict of interest.

## Footnotes

^1^http://www.episoft.co.nz/iris

^2^https://www.R-project.org/

^3^http://www.nait.co.nz/

^4^https://www.foodsafety.govt.nz/elibrary/industry/animal-status-declaration/
